# Adaptive hindlimb split-belt treadmill walking in rats by controlling basic muscle activation patterns via phase resetting

**DOI:** 10.1038/s41598-018-35714-8

**Published:** 2018-11-26

**Authors:** Soichiro Fujiki, Shinya Aoi, Tetsuro Funato, Yota Sato, Kazuo Tsuchiya, Dai Yanagihara

**Affiliations:** 1 Department of Life Sciences, Graduate School of Arts and Sciences, The University of Tokyo, 3-8-1 Komaba, Meguro-ku, Tokyo 153-8902 Japan; 2 Department of Aeronautics and Astronautics, Graduate School of Engineering, Kyoto University, Kyoto daigaku-Katsura, Nishikyo-ku, Kyoto 615-8540 Japan; 3 Department of Mechanical Engineering and Intelligent Systems, Graduate School of Informatics and Engineering, The University of Electro-communications, 1-5-1 Chofugaoka, Chofu-shi, Tokyo 182-8585 Japan

## Abstract

To investigate the adaptive locomotion mechanism in animals, a split-belt treadmill has been used, which has two parallel belts to produce left–right symmetric and asymmetric environments for walking. Spinal cats walking on the treadmill have suggested the contribution of the spinal cord and associated peripheral nervous system to the adaptive locomotion. Physiological studies have shown that phase resetting of locomotor commands involving a phase shift occurs depending on the types of sensory nerves and stimulation timing, and that muscle activation patterns during walking are represented by a linear combination of a few numbers of basic temporal patterns despite the complexity of the activation patterns. Our working hypothesis was that resetting the onset timings of basic temporal patterns based on the sensory information from the leg, especially extension of hip flexors, contributes to adaptive locomotion on the split-belt treadmill. Our hypothesis was examined by conducting forward dynamic simulations using a neuromusculoskeletal model of a rat walking on a split-belt treadmill with its hindlimbs and by comparing the simulated motions with the measured motions of rats.

## Introduction

Locomotor adaptability in animals has been investigated using a split-belt treadmill, which has two parallel belts whose speeds are controlled independently to prepare left–right symmetric and asymmetric environments for walking^1–10^. When the configuration of the treadmill changes from the tied (two belts move at the same speed) to the split-belt configuration (two belts move at different speeds), locomotion parameters, such as the relative phase between the legs and duty factors, change to maintain walking^6,8^. Frigon *et al*.^2^ reported that chronic spinal cats and intact cats showed similar adaptive locomotor behavior on a split-belt treadmill even though the spinal cord of the spinal cats had been transected from the brain. This indicates that the spinal cord and associated peripheral nervous system contributed to the adaptive locomotion.

Physiological studies to date have suggested that a neural network called a central pattern generator (CPG) in the spinal cord contributes greatly to the generation of adaptive locomotion^11,12^. In particular, while the CPG can produce rhythmic signals without sensory feedback in a feedforward manner, the sensory feedback is crucial to achieve adaptive locomotion^13^. Although the sensorimotor coordination mechanism remains unclear, it has been suggested that the CPG uses a low dimensional structure related to muscle synergies for the sensorimotor coordination. More specifically, because animals have redundant musculoskeletal systems, they have to solve the redundancy problem for motor control. Ivanenko *et al*.^14,15^ showed that the linear combination of only a few basic temporal patterns accounts for most of the electromyography (EMG) data measured during locomotion and suggested that the CPG produces a few pulses for one gait cycle that are distributed to motoneurons to create motor commands. Furthermore, the onset timing of the basic patterns is strictly linked to specific kinematic events, such as liftoff of the feet, suggesting that the CPG manipulates the onset timings of the pulses based on specific sensory feedback. It has been reported that the sensory signals from the ankle extensor and hip flexor muscles contribute to the timing regulation of motor commands. In particular, when the force feedback from ankle extensor is less than the lower limit, the flexor muscle activity was initiated^16–18^. When the hip flexor muscles are stretched, ongoing antagonistic extensor activities are interrupted, and flexor activities are initiated to raise the foot for swinging of the leg^16,18,19^. That is, the locomotor phase is reset (phase resetting) to regulate the stance-to-swing transition. However, it remains unclear to what extent such sensory feedbacks contribute to locomotor adaptability.

One method to investigate the functional role of a specific part of the nervous system is to examine dysfunction of the nervous system. However, because the nervous system is intricately organized, dysfunction of one part of the nervous system influences the functions of other parts of the nervous system. Consequently, a vast amount of physiological evidence for identifying the functional role of a part of the nervous system must be accumulated. In addition, to clearly show the correlation or causation between neural activity and the motor output, it is necessary to measure them simultaneously. However, it is difficult to measure the activity of neurons during dynamic motor tasks. To overcome such limitations, modeling studies have attracted attention^18,20–29^. Because locomotion is well-organized behavior generated through dynamic interactions between the neural system, musculoskeletal system, and environment, it is crucial to develop each model and then integrate them. Physiological findings and hypotheses allow us to create reasonably realistic neural models, and anatomical and biomechanical findings allow us to construct musculoskeletal models.

In this study, our working hypothesis was that resetting the onset timings of basic temporal patterns based on the sensory information, such as extension of hip flexors, contributes to adaptive split-belt treadmill walking. Our hypothesis was examined by conducting forward dynamic simulations using a neuromusculoskeletal model of a rat with its hindlimbs. Because rats have been used as subjects for numerous physiological experiments^13,30–32^, various findings have already been accumulated. Moreover, because in recent years genetic modification technologies have been applied to rats, they are expected to be subjects in a much wider range of physiological experiments^33–35^. For the nervous system model to examine our hypothesis, a motor control and sensorimotor integration model was constructed based on the physiological concept of the CPG. Markin *et al*.^25^ used a physiologically detailed Hodgkin-Huxley (HH) type neuron model and sensory feedback signals from Ia, II, and Ib sensory fibers, and cutaneous receptors for cat hindlimb walking. In contrast, in the present study, simple CPG model using phase oscillators was used to more simply understand the locomotor adaptation mechanism from the perspective of dynamics, based on the muscle synergy hypothesis and phase resetting by sensory feedback signals from the legs. More specifically, the CPG model produces motor commands by the linear combination of a few pulses and manipulates the activation timing of the pulses through phase resetting based on hip extension. Forward dynamic simulations were performed to investigate whether control of the activation timing of a few pulses through phase resetting based on hip extension induced adaptive locomotor behavior during bipedal split-belt treadmill walking. That is, how locomotor adaptation appears was examined through dynamic interactions between the nervous system, musculoskeletal system, and split-belt treadmill environment models. In addition, hindlimb split-belt treadmill walking of rats was measured in the present study, and the simulation results were compared with the measured data. Furthermore, the contribution of the timing control of a few pulses by phase resetting to locomotor adaptability is discussed.

## Results

### Joint kinematics during split-belt walking

**Figure 1:**
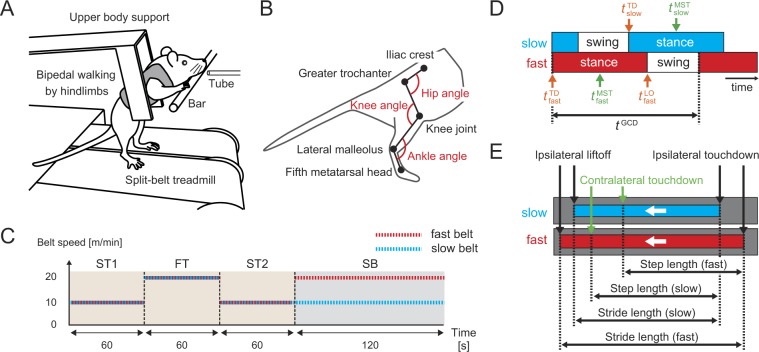
Experimental setup and major locomotion parameters. (**A**) Rat walking on split-belt treadmill by the hindlimbs. The rats rested their forepaws on the bar in front of them and wore a harness connected with a flexible beam fixed on the treadmill. (**B**) Marker positions and definitions of angles. (**C**) Experimental procedure of one session (2.0x condition), where the two belts moved at 10 m/min in the ST and at 20 m/min in the FT, and one belt moved 2.0 times faster than the other belt moving at 10 m/min during the SB. ST: slow-tied configuration, FT: fast-tied configuration, SB: split-belt configuration. (**D**) Temporal parameters. ${t}_{i}^{{\rm{TD}}}$, ${t}_{i}^{{\rm{LO}}}$, and ${t}_{i}^{{\rm{MST}}}$ (*i* = fast, slow) are the time at touchdown, liftoff, and middle of stance phase, respectively. *t*^GCD^ is the gait cycle duration. Relative phase between the two legs is calculated by $2{\rm{\pi }}({t}_{{\rm{fast}}}^{{\rm{MST}}}-{t}_{{\rm{slow}}}^{{\rm{MST}}})/{t}^{{\rm{GCD}}}$. Duty factor is calculated by the ratio of the stance phase relative to the gait cycle duration. Liftoff timing of the fast leg and touchdown timing of the slow leg relative to touchdown timing of the fast leg is calculated by $2{\rm{\pi }}({t}_{{\rm{fast}}}^{{\rm{LO}}}-{t}_{{\rm{fast}}}^{{\rm{TD}}})/{t}^{{\rm{GCD}}}$ and $2{\rm{\pi }}({t}_{{\rm{slow}}}^{{\rm{TD}}}-{t}_{{\rm{fast}}}^{{\rm{TD}}})/{t}^{{\rm{GCD}}}$, respectively. (**E**) Spatial parameters (stride length and step length). Stride length is defined by the distance from the touchdown position to the liftoff position of the foot for each leg. Step length is defined by the distance between the foot positions of the two legs at the touchdown of one leg along the direction of belt movement (fast/slow step length means the step length at the touchdown of the fast/slow leg). White arrows show the moving direction of the belts.

The motions of the walking on a split-belt treadmill in rats were measured by 3D motion capture system (Fig. 1A,B). The experimental procedure was based on previous studies^4–8^ and Fig. 1C shows the experimental procedure in one session, which includes slow-tied configuration, fast-tied configuration, and split-belt configuration. A speed of 10 m/min was used for the slow belt speed in the slow-tied and split-belt configurations, and speeds of 15, 17, or 20 m/min were used for the fast belt speed in the fast-tied and split-belt configurations (1.5x, 1.7x, and 2.0x conditions). The number of sessions performed was shown in Table 1. The leg on the fast belt in the split-belt configuration was called the fast leg, and the leg on the slow belt was called the slow leg.

**Table 1:** Belt speed ratio and number of sessions used for statistical analysis.

Speed ratio	Number of sessions (fast side)	
	Analysis of locomotion parameters and transition timings	Analysis of hip angle at liftoff
1.5x	15 (right 10, left 5)	13 (right 8, left 5)
1.7x	12 (right 5, left 7)	14 (right 7, left 7)
2.0x	11 (right 2, left 9)	11 (right 2, left 9)

The speed ratio shows the ratio between the belt speeds (fast/slow) used in the split-belt configuration. Data in the sessions are distributed to two analysis groups based on the marker positions for calculation (the marker on the fifth metatarsal head was used for locomotion parameters and transition timings, and the marker on the iliac crest was used for the hip angle). Number of sessions represents the total number of data sets used for each analysis, where fast side shows which side (right or left) was used for the fast belt during the split-belt configuration and how many sessions were conducted for the side. Some data were excluded from the analysis because the markers were not sufficiently captured. Detailed information is shown in Supplementary Table S4.

**Figure 2:**
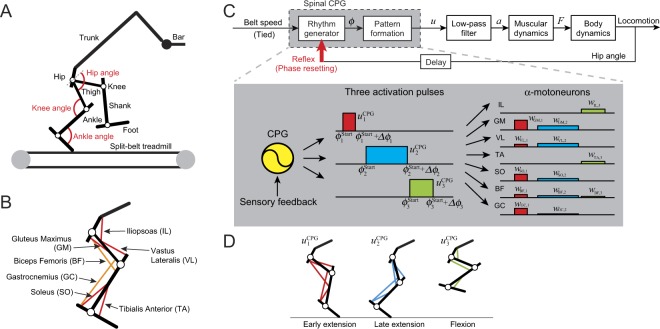
Neuromusculoskeletal model of rat. (**A**) Skeletal model on split-belt treadmill. (**B**) Muscle model. (**C**) Schematics of our model. A CPG produces a motor command by a combination of three activation pulses and manipulates activation timing by sensory feedback. (**D**) Muscles activated by each pulse.

Our previous neuromusculoskeletal model of rat hindlimbs used for overground walking and obstacle avoidance^20^ was modified to apply it to split-belt treadmill walking in the present study. Figure 2A,B showed the musculoskeletal model of a rat on a split-belt treadmill (see Supplementary Method S2). The spinal CPG model, for which a phase oscillator was used in each limb, produce three activation pulses depending on its phase and the motor commands were determined by the linear combination of these pulses (Fig. 2C). Three activation pulses contributed to early extension, late extension, and flexion phases, respectively (Fig. 2D). Sensory information about the stretch of the hip flexor muscle was used for the sensory feedback model (equation (5)). This feedback model reset the phase of the CPG model to the onset phase of third activation pulse (phase resetting). The same speed condition for the belts was used as the measurement of rats (1.5x, 1.7x, and 2.0x). To investigate the locomotor adaptability of the model, only the slow-tied and split-belt configurations were used for the simulation study, and the belt speed condition was suddenly changed from the slow-tied to split-belt configurations. When the belt speed condition was changed, no control parameters of the model were changed, and if the rat model kept walking and how the locomotor behavior changed were examined. To clearly show the contribution of the phase resetting to gait adaptation, the cases with and without phase resetting [the rat model with phase resetting uses equation (5) while the model without phase resetting uses equation (1)] were compared. The control parameters were determined so that the rat model with and without phase resetting showed similar stable periodic gait (limit cycle) in the slow-tied configuration.

**Figure 3:**
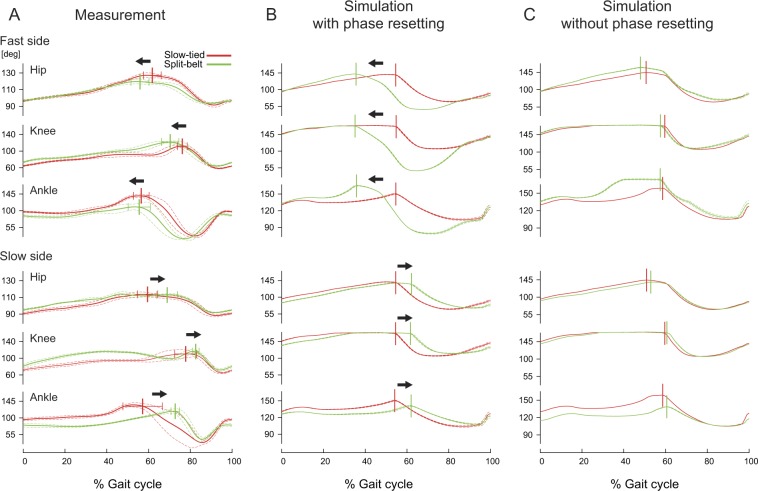
Comparison of joint kinematics. Comparison of joint angles for fast and slow sides during each period [slow-tied configuration and split-belt configuration] between measurements of rats (**A**), simulation with phase resetting (**B**), and simulation without phase resetting (**C**). Solid and dashed lines represent the average and standard error for over 10 steps, respectively. Vertical lines and error bars represent the average and standard deviation of the peak timings for over 10 steps. 0 and 100% indicate touchdown timing. A shows representative results. A and B used the 2.0x condition, and C used the 1.7x condition. See Supplementary Movies (S1, S2 and S3) for the simulated locomotor behaviors.

Figure 3 compares the profiles of joint angles for both sides during the slow-tied and split-belt configurations for (A) the measurements of rats, (B) simulation with phase resetting, and (C) simulation without phase resetting. Figure 3A shows representative results for the split-belt configuration using the 2.0x condition (Session No. 35, Supplementary Table S4). In the fast side, the peak phases of the joint angles of the split-belt configuration came earlier than those of the slow-tied configuration due to the speed increase. In contrast, the peak phases in the slow side came later than those of the slow-tied configuration, even though the belt speeds were identical. This change in the peak phases was observed in the rats in most cases. Although the model with phase resetting kept walking at the split-belt configuration even in the 2.0x condition, the model without phase resetting could not continue walking and easily fell down after the change in the belt speed condition. It kept walking only up to the 1.7x condition. The model with phase resetting and the measurements of rats showed a similar trend in the peak phases of the joint angles (Fig. 3B). On the other hand, the peak phases did not change very much in the model without phase resetting because this model could not change the generation timings of the activation pluses in accordance with sensory feedback (Fig. 3C). See Supplementary Movies (S1, S2, and S3) for the simulated locomotor behaviors.

### Major locomotion parameters

**Figure 4:**
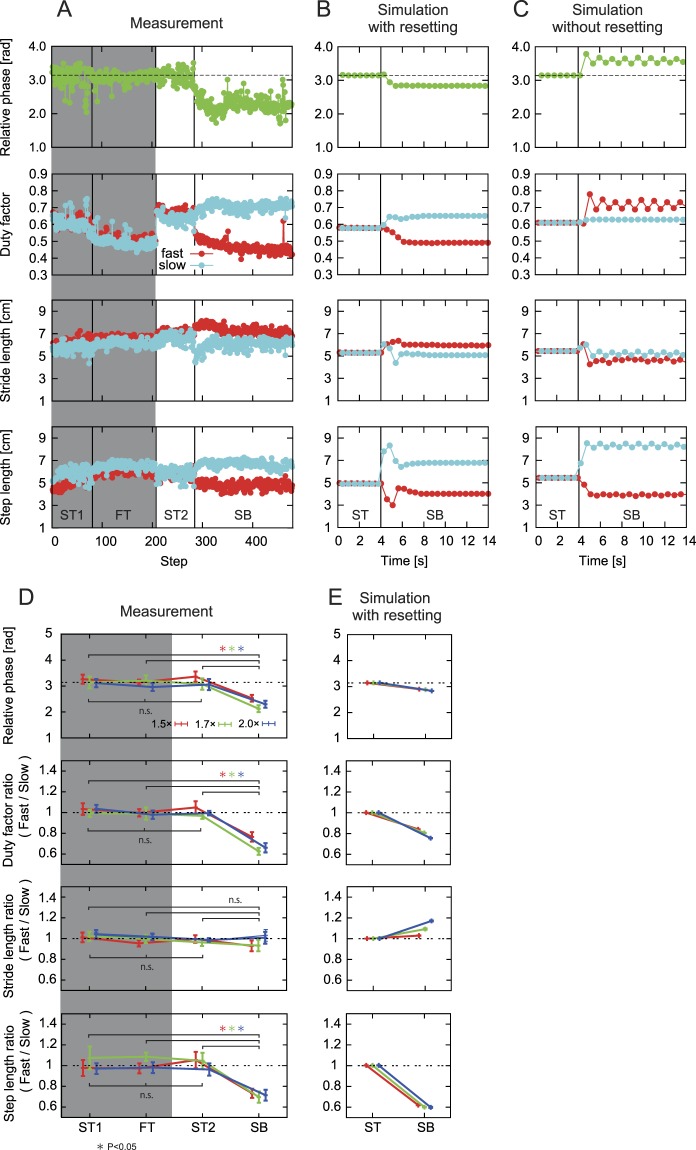
Comparison of four locomotion parameters (relative phase, duty factor, stride length, and step length). (**A**–**C**) Comparison of the time course during the slow-tied configuration (ST) and split-belt configuration (SB) among measurements of rats (**A**), simulation with phase resetting (**B**), and simulation without phase resetting (**C**). A shows representative results, with the results during the first ST (ST1) and fast-tied configuration (FT) for reference (gray region). A and B used the 2.0x condition, and C used the 1.7x condition. D, E: Comparison of the averages during the ST and SB with three belt speed conditions (1.5x, 1.7x, and 2.0x) between measurements of rats (**D**) and simulation with phase resetting (**E**). In D, data points and error bars are the average and standard error values between the sessions in each belt speed condition. In E, data points and error bars are the average and standard error values for more than five steps in each belt speed condition.

Based on previous studies^6,8^, the four major locomotion parameters (relative phase between the two legs and duty factor are related to temporal characteristics, and stride length and step length are related to spatial characteristics, Fig. 1D,E) were calculated. Figure 4A–C compare the time profiles of the four major locomotion parameters during the slow-tied and split-belt configurations among (A) the measurements of rats, (B) simulation with phase resetting, and (C) simulation without phase resetting. Figure 4A shows representative results (Session No. 31, Supplementary Table S4) for the split-belt configuration using the 2.0x condition, with the results during the slow-tied 1 and fast-tied configurations for reference (gray region). The relative phase between the legs was almost anti-phase during the slow-tied configuration and shifted downward during the split-belt configuration. The duty factor, stride length, and step length were almost identical between the slow and fast legs during the slow-tied configuration. While the duty factor and step length of the slow leg increased, and those of the fast leg decreased during the split-belt configuration, the stride length of the slow leg decreased, and that of the fast leg increased. The changes in the stride length were small relative to the other three parameters. These trends of the locomotion parameters were observed in the rats in most cases. The model with phase resetting and the measurement of rats showed similar trends in these four locomotion parameters (Fig. 4B). Although the model without phase resetting and the measurements of rats showed a similar trend in the step length, the model without phase resetting showed different trends in the relative phase, duty factor, and stride length during the split-belt configuration (Fig. 4C). Furthermore, these locomotion parameters fluctuated during the split-belt configuration in the model without phase resetting, in contrast to the stable convergence in the model with phase resetting.

To examine if there were differences in locomotor behavior between the slow side and the fast side depending on the configuration of the treadmill, one-way repeated measures analysis of variance (ANOVA) was used for four locomotion parameters obtained from the measured data of rats among the four testing periods (slow-tied 1, fast-tied, slow-tied 2, and split-belt configurations) for each speed condition (1.5x, 1.7x, and 2.0x). Figure 4D,E compare the average values of the four locomotion parameters between (D) the measurement of rats and (E) the simulation with phase resetting. Figure 4D also shows the results during the slow-tied 1 and fast-tied configurations for reference (gray region). During the slow-tied 1, fast-tied, and slow-tied 2 configurations, the relative phase between the legs was almost anti-phase (Fig. 4D). During the split-belt configuration, it decreased from almost anti-phase. During the slow-tied 1, fast-tied, and slow-tied 2 configurations, the fast/slow ratio of the duty factor and the fast/slow ratio of the step length were almost 1. During the split-belt configuration, they decreased significantly. The fast/slow ratio of the stride length was almost 1 during the whole periods and did not show any significant changes. The detailed results of the analysis are shown in Supplementary Table S1. The model with phase resetting and the measurements of rats showed qualitatively similar trends (Fig. 4E).

### Liftoff and touchdown timings

**Figure 5:**
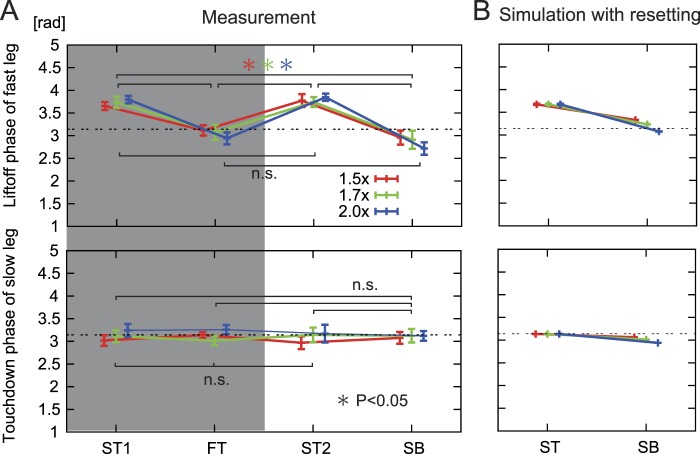
Comparison of liftoff and touchdown phases. Comparison of the liftoff phase of the fast leg and touchdown phase of the slow leg during the slow-tied configuration (ST) and split-belt configuration (SB) in each belt speed condition (1.5x, 1.7x, and 2.0x) between the measurements of rats (**A**) and simulation with phase resetting (**B**). In A, data points and error bars are the average and standard error values between the sessions in each belt speed condition with the results during the first ST (ST1) and fast-tied configuration (FT) for reference (gray region). In B, data points and error bars are the average and standard error values for more than five steps in each belt speed condition.

To produce adaptive locomotor behavior depending on the environmental situation, adaptive transition of the controls for the stance and swing phases is crucial^36^. To regulate the transition timing, liftoff and touchdown timings are important. Based on a previous study^4^, how these timings changed depending on the belt speed condition was investigated. Figure 5 compares the liftoff phase of the fast leg and the touchdown phase of the slow leg based on the touchdown phase of the fast leg between (A) the measurements of rats and (B) the simulation with phase resetting. Figure 5A also shows the results during the slow-tied 1 and fast-tied configurations for reference (gray region). To examine if the timings of the swing-to-stance and stance-to-swing transitions varied depending on the configuration of the treadmill, one-way repeated measures ANOVA was used for liftoff and touchdown timings obtained from the measured data of rats among the four testing periods (slow-tied 1, fast-tied, slow-tied 2, and split-belt configurations) for each speed condition (1.5x, 1.7x, and 2.0x). The liftoff phase of the fast leg was almost 4 rad during the slow-tied configuration and decreased significantly to almost π rad during the split-belt configuration. On the other hand, the touchdowns of the two legs continued to alternate (almost π rad relative phase) in the split-belt experiments. ANOVA did not show significant changes between testing periods. The detailed results of the analysis are shown in Supplementary Table S2. The model with phase resetting and the measurements of rats showed similar trends (Fig. 5B).

**Figure 6:**
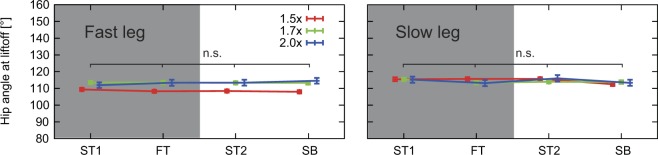
Comparison of hip joint angles at liftoff. Hip joint angles at liftoff of the fast and slow legs during the slow-tied configuration (ST), fast-tied configuration (FT), and split-belt configuration (SB) in each belt speed condition (1.5x, 1.7x, and 2.0x) in the measurements of rats. Data points and error bars are the average and standard error values between animals in each belt speed condition. One-way repeated measures analysis of variance (ANOVA) did not show significant differences among the four testing periods.

The liftoff timing depended on the environmental situation (Fig. 5). The stance-to-swing phase transition occurs when the hip joint angle exceeds a threshold angle, and it has been suggested that sensory signals related to the hip extension contribute to the liftoff timing^37^. Based on this hypothesis, the reflexive regulation of motor commands was modeled by equation (5) (phase resetting based on the hip joint angle). To verify this hypothesis and the validity of our model, the hip joint angle at liftoff was investigated from the measured data of rats. Figure 6 shows the average hip angles at liftoff of the fast and slow legs between the sessions during the four periods in each belt speed condition. The error bars represent the standard errors. To examine if hip extension varied at the stance-to-swing transition, one-way repeated measures ANOVA was used for hip joint angles at liftoff obtained from the measured data of rats among the four testing periods (slow-tied 1, fast-tied, slow-tied 2, and split-belt configurations) for each speed condition (1.5x, 1.7x, and 2.0x). ANOVA did not show any significant differences in the fast leg and in the slow leg (Supplementary Table S3). This result suggests that the rats lifted up their legs when the hip joint angle reached a certain value regardless of the belt speed condition of the treadmill, as used in our model.

**Figure 7:**
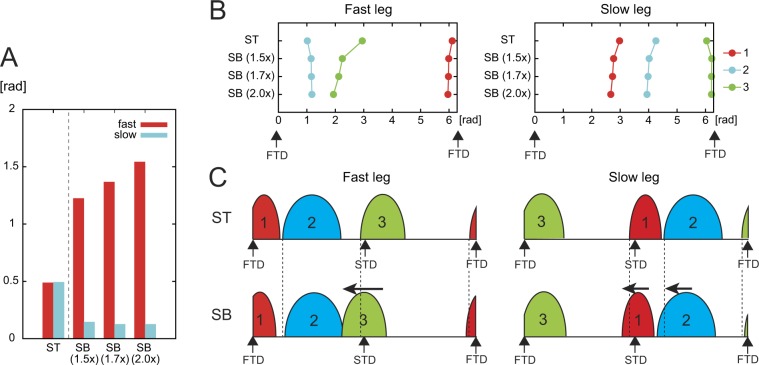
Contribution of phase resetting to manipulate the onset timing of the activation pulses in our model. (**A**) Values of phase resetting during the slow-tied configuration (ST) and split-belt configuration (SB) in three belt speed conditions (1.5x, 1.7x, and 2.0x). Positive values mean that the phase is shifted in the forward direction. (**B**) Activation phase of the three activation pulses of the fast and slow legs based on the touchdown phase of the fast leg (FTD). (**C**) Schematic illustration of the change in onset timings of the pulses from the ST to SB. STD: touchdown of the slow leg.

In our model, the motor command was determined by the linear combination of a few activation pulses, and phase resetting manipulated the onset timing of the activation pulses based on the hip joint angle, which allowed the rat model to achieve adaptive split-belt treadmill walking whose characteristics were similar to those in rats. To see how the phase resetting contributed to the adaptive locomotion in our model, the value of resetting was investigated in Fig. 7 (positive values mean that the oscillator phase is shifted in the forward direction). From the slow-tied to split-belt configurations, the values of resetting increased in the fast leg and decreased in the slow leg as shown in Fig. 7A (time profiles of the oscillator phases in the 2.0x condition are shown in Supplementary Fig. S1). These changes increased as the belt speed condition changed, especially in the fast leg. This increase in the fast leg was caused by the increase of the fast belt speed. The hip joint angle of the fast side exceeded the threshold earlier, and the oscillator phase of this side was shifted more in the forward direction. Figure 7B,C show how onset timing of the activation pulses varied by phase resetting based on the locomotion phase calculated by the touchdown of the fast leg. In particular, activation pulse 3, which contributes to the flexion of the leg, became much earlier in the split-belt configuration in the fast leg. Furthermore, through the interaction between the oscillators (second term in equation (5)), activation pulses 1 and 2 of the contralateral leg (slow leg) became earlier. The magnitude of these changes increased as the belt speed increased.

## Discussion

In the present study, the adaptive locomotion mechanism during split-belt treadmill walking was investigated using a neuromusculoskeletal model of rats. In this model, the focus was on the functional roles of the spinal cord and peripheral nervous system, especially hip extension for the stance-to-swing transition, to achieve adaptive locomotion for the changes in the belt speed condition. More specifically, a low dimensional control strategy was used to generate periodic motor commands based on the muscle synergy hypothesis, and a phase resetting mechanism by the afferent signals about the hip extension was used to modulate the phase of the motor commands immediately. The simulation results were compared with the measured data in rats, which showed that the adaptation trends of the model with phase resetting were qualitatively similar to those of the rats. In contrast, the locomotor behavior of the model without phase resetting was different from that measured during the split-belt configuration. This suggests that hip extension contributed to adaptive locomotion on the split-belt treadmill.

Animals produce appropriate interlimb and intralimb coordination to adapt to their postural disturbances and environmental changes. In particular, in the split-belt treadmill walking for rats, duty factor and stride length showed adaptive intralimb coordination, and relative phase between the legs and step length showed adaptive interlimb coordination (Fig. 4). Because gait is generated by the leg controls for the swing and stance phases, adaptive transitions of these controls are crucial. It has been suggested that the CPG in the spinal cord contributes to the adaptive transitions with peripheral sensory information^2,38^. One of the well-known pieces of peripheral sensory information is kinematics (muscle length) and the CPG program is suggested to contain critical points at phases corresponding to important kinematic events in locomotion^39–41^. When proprioceptive perturbations were applied at these critical points, the onset of a segment of EMG shifted from its own critical phase to an earlier or later critical point. Furthermore, when chronic spinal cats walking on a treadmill were intercepted so that the leg motions were forced to stop and to move backward gradually, the onset of the swing phase was observed when the hip angle exceeded a threshold angle^37^. The threshold angle was almost identical to that in normal walking. This suggests that the hip angle information was used to determine the onset of the swing motion. In split-belt treadmill walking, the fast leg is pulled faster than the slow leg in the split-belt configuration and the hip joint of the fast leg is extended more quickly. To adapt to this asymmetric locomotor condition, rats produced earlier liftoff phases in the fast leg (Fig. 5) without changing the hip joint angle at liftoff (Fig. 6). The present study focused on these observations, and the hip angle information was used for the peripheral information in our model. The simulation results showed that immediate phase modulation based on the hip joint angle to initiate the swing motion by phase resetting allowed the model to achieve adaptive locomotor behaviors, whose characteristics were similar to those of the rats (Figs 3, 4 and 5). The model without the phase modulation could not obtain appropriate flexion timings in the fast leg in the split-belt configuration. The fast leg was pulled for a longer time, and the hip joint was greatly extended. This increased the stance phase duration (duty factor) in the fast leg, which caused different locomotor characteristics, especially in the temporal elements from the measurements in rats and the model with phase modulation (Figs 3, 4 and 5). Moreover, as the fast leg was moved away from the center of gravity, it became more difficult to recover from the postural disturbance. These factors resulted in walking that was less robust. Although the model with the phase modulation could continue to walk even in the 2.0x condition, the model without the phase modulation could only walk up to the 1.7x condition. As well as the stance-to-swing transition, the swing-to-stance transition is also important^42^. However, because the touchdown phases of the slow leg were not altered in the split-belt configuration unlike the liftoff phases of the fast leg in rats (Fig. 5), the phase modulation was incorporated in our model only at the stance-to-swing transition. The contribution of the adaptive swing-to-stance transition will be investigated in future studies.

Peripheral signals other than those related to the hip joint angle also contribute to adaptive locomotion. For example, increasing the load on the ankle extensor muscle in decerebrate cats during walking decreased the duration of ankle flexor muscle activities^16,17^. When the load exceeded a threshold, the flexor muscle showed no activity. Similar phenomena were observed in intact cats^43^, humans^44^, and rats^30^, and it is suggested that sensory information related to the load on the ankle extensor muscle contributes to the initiation of the swing phase. In spinal cats, electrical stimulation of the sural cutaneous nerve during the swing phase increases the duration of the swing motion, and that during the stance phase induces the transition to the swing phase^45^. Stimulation of the dorsum of the paw increases flexor activities during the swing phase^46^. These results suggest that cutaneous sensory information contributes to the regulation of leg motion. Cat hindlimb models were used to demonstrate the effects on walking stability of sudden removal of the length feedback from the hip muscles and force feedback from ankle extensors^18,25^. The contributions of the different sensory information to adaptive locomotion in our model will be investigated in future studies. Moreover, for more detailed understanding the phase resetting mechanism for these sensory feedbacks, the mathematical analysis of the neural dynamics including isochrons and phase response curves based on neuron model would be helpful^47^.

Multiple muscles were activated cooperatively depending on the motor task. Shinoda *et al*.^48^ traced one axon in the medial vestibulospinal tract and showed that the axon projected to multiple motoneurons for neck muscles. Although it is suggested that descending tracts from the brain to the spinal cord affect motoneuronal pools of multiple muscles via spinal interneurons, the anatomical structure of other parts of the body to control multiple muscles remains unclear. Recently, many studies have extracted coordinated structures in muscle activations (muscle synergy) from the EMG data of many muscles using mathematical operations, such as principle component analysis, factor analysis, and non-negative matrix factorization^14,15,31,49–58^. For locomotion, most of the EMG data are accounted for by the linear combination of a few basic patterns. The extracted low-dimensional coordination structure was discussed not on an anatomical basis, but in terms of motor function. It has been suggested that the CPG in the spinal cord was composed of rhythm generation layer and pattern formation layer^59^ and that neurons’ activities in the pattern formation layer contribute to the muscle synergy structure (topographic map for muscle synergy)^60^. Three basic patterns extracted from the EMG data of bipedally walking rats on a treadmill contributed to early extension, late extension, and flexion, respectively, of the leg^31^, as used in our model (Fig. 2D). The comparison of the motoneuron activities during fictive locomotion in decerebrate cats with the muscle activities during real locomotion in intact cats suggested that afferent feedback from most monoarticular muscles controls only the timing of the flexion–extension or extension–flexion phase transitions and the durations of their phases^55^. Our phase regulation model with phase resetting by sensory feedback allowed the control of each activation pulse in accordance with the environmental situation (Fig. 7B,C) (such a phase regulation of basic patterns has been observed in human split-belt treadmill walking^4^). This model achieved characteristics similar to those of rats in adaptive locomotor behaviors (Figs 3, 4 and 5) and robust locomotion (Figs 3 and 4). This suggests that the sensory regulation model captures the essential aspect of adaptive motor control in walking. Although we focused on the rhythm generation layer for the adaptation in the spit-belt treadmill walking, the pattern formation layer might also have an important contribution to the adaptation, such as topographic map of muscle synergy^60^. In future work, we would like to investigate the contribution by improving our model.

In split-belt treadmill walking, locomotor behaviors immediately varied by changing the belt speed condition. In healthy humans, gradual changes in specific locomotion parameters related to interlimb coordination appear after the immediate changes, and an aftereffect appears after the belt speed returns to the original condition^8^. It has been reported that patients with cerebellar damage show immediate changes in locomotion parameters, but showed neither the gradual changes nor aftereffects^6^. It was suggested that the gradual changes and aftereffects were induced by the effect of motor learning for which the cerebellum is responsible. In the gradual modulation in human split-belt treadmill walking, ankle stiffness is modulated^7^ and the left–right asymmetry of vertical ground reaction forces at foot touchdown is reduced^5^. These results suggest that the swing-to-stance transition would be key for the gradual modulation. In our previous study^61^, we developed a locomotor control system for a biped robot, which regulated the swing-to-stance transition timing through learning. The robot experiments showed gradual changes of locomotion parameters, whose trends were similar to those of humans. In future studies, we would like to improve our neuromusculoskeletal model of rats by incorporating a motor learning model to examine the underlying mechanism for the gradual changes and aftereffects observed in adaptive locomotor behavior.

## Methods

### Measurements on rats

#### Experimental procedure

This study was approved by the Ethical Committee for Animal Experiments at the University of Tokyo and conducted in accordance with the Guidelines for Research with Experimental Animals of the University of Tokyo.

Thirteen intact male Wistar rats [183 ± 8 g (body mass ± standard deviation)] were used in the experiment. A custom-made split-belt treadmill was used. The length and width of each belt were 20 and 10 cm, respectively. The rats rested their forepaws on the bar in front of them and wore a harness connected with a flexible beam fixed on the treadmill. The harness supported their upper body so that more than half of their weight was put on their hindlimbs and the hindlimb motions were not encumbered. Prior to the measurements, the rats were trained to walk bipedally by their hindlimbs in the tied configuration. During the training and measurements, 4% sucrose water was used as a reward, and the rats could drink it freely through a tube in front of them (Fig. 1A).

Based on the experimental procedures in previous studies^4–8^, the following four speed conditions were used for one session (Fig. 1C): 1. slow-tied configuration for 60 s, where both belt speeds were slow; 2. fast-tied configuration for 60 s, where both belt speeds were fast; 3. slow-tied configuration for 60 s; and 4. split-belt configuration for 120 s, where one belt speed was slow and the other was fast. After each speed condition, the belts were stopped, and then the next condition was started within 1 min. The leg on the fast belt in the split-belt configuration was called the fast leg, and the leg on the slow belt was called the slow leg. A speed of 10 m/min was used for the slow belt speed in the slow-tied and split-belt configurations. In contrast, speeds of 15, 17, or 20 m/min were used for the fast belt speed in the fast-tied and split-belt configurations, depending on the session (1.5x, 1.7x, and 2.0x conditions). The number of sessions performed was shown in Table 1. The rat motions were measured by a 3D motion capture system (Qualisys, Gothenburg, Sweden) (see Supplementary Method S1).

#### Definition of locomotion parameters

Based on previous studies^6,8^, we calculated following seven locomotion parameters to investigate adaptation.

Relative phase between the left and right hindlimbs, which is calculated by 2 ${\rm{\pi }}({t}_{{\rm{fast}}}^{{\rm{MST}}}-{t}_{{\rm{slow}}}^{{\rm{MST}}})/{t}^{{\rm{GCD}}}$, where ${t}_{{\rm{fast}}}^{{\rm{MST}}}$ and ${t}_{{\rm{slow}}}^{{\rm{MST}}}$ are the times at the middle of the stance phase for the fast leg and slow leg, respectively, and *t*^GCD^ is the gait cycle duration.

Duty factor, which is the ratio of the stance phase duration relative to one gait cycle.

Stride length, which is the distance from the touchdown position to the liftoff position of the foot for each leg.

Step length, which is the distance between the foot positions of the two legs at the touchdown of one leg along the direction of belt movement (fast/slow step length means the step length at the touchdown of the fast/slow leg).

The liftoff timing of the fast leg and the touchdown timing of the slow leg were defined relative to the touchdown timing of the fast leg by $2{\rm{\pi }}({t}_{{\rm{fast}}}^{{\rm{LO}}}-{t}_{{\rm{fast}}}^{{\rm{TD}}})/{t}^{{\rm{GCD}}}$ and 2 ${\rm{\pi }}({t}_{{\rm{slow}}}^{{\rm{TD}}}-{t}_{{\rm{fast}}}^{{\rm{TD}}})/{t}^{{\rm{GCD}}}$, respectively, where ${t}_{{\rm{fast}}}^{{\rm{LO}}}$ and ${t}_{{\rm{fast}}}^{{\rm{TD}}}$ are the times at the liftoff and touchdown of the fast leg, and ${t}_{{\rm{slow}}}^{{\rm{TD}}}$ is the time at the touchdown of the slow leg (Fig. 1D), as in a previous study^4^.

Hip joint angles at liftoff was calculated to investigate the contribution of the sensory information by hip extension.

#### Statistical analysis

For statistical analysis, the data measured during 10 steps after the rats started walking in each period (slow-tied 1, fast-tied, slow-tied 2, and split-belt configurations) were used (Table 1). The average values for the relative phase between the two legs, liftoff and touchdown timings, and hip joint angles at liftoff were used. In contrast, the ratios of the average values for the duty factor, stride length, and step length were calculated to investigate the differences between the slow and fast sides. When the ratio was 1, there was no difference between the fast and slow sides for the parameters. To examine if there were differences in locomotor behavior depending on the configuration of the treadmill, one-way repeated measures analysis of variance (ANOVA) was used for the locomotion parameters among the four testing periods (slow-tied 1, fast-tied, slow-tied 2, and split-belt configurations) for each speed condition (1.5x, 1.7x, and 2.0x). When the ANOVA showed a significant difference, post hoc analysis was performed using Tukey’s honestly significant different test. Some data were excluded from the statistical analysis, because some markers were not captured during the sessions, as shown in Table 1.

### Modeling

#### Musculoskeletal model

Rat hindlimb muscloskeletal model, which included seven muscles for each hindlimb ([iliopsoas (IL), gluteus maximus (GM), vastus lateralis (VL), tibialis anterior (TA), soleus (SO), biceps femoris (BF), and gastrocnemius (GC)]), was based on our previous work^20^ (See Supplementary Method S2). The same speed condition for the belts as used for the measurement of rats was used. In contrast to the measurement of rats, only the slow-tied and split-belt configurations were used for the simulation study, and the belt speed condition was suddenly changed from the slow-tied to split-belt configurations to examine if the rat model kept walking and how the locomotor behavior changed. Forward dynamic simulations were performed by solving the governing equations using a fourth-order Runge–Kutta method with a step size of 0.02 ms.

#### Nervous system model

The nervous system model consisted of two parts (Fig. 2C): 1. limb movement control by the combination of a few activation pulses in a feedforward fashion based on the muscle synergy hypothesis; and 2. sensory reflex to regulate the activation timing of the pulses through phase resetting.

Although the organization of the CPG remains unclear, it has been suggested that the CPG consists of a two-layered network composed of the rhythm generator (RG) network, which produces rhythm and phase information for motor commands, and the pattern formation (PF) network, which produces spatiotemporal patterns of motor commands and sends them to the motoneurons^59^. Moreover, it has been suggested that synaptic connections exchanging information about left–right and fore–hind limbs are in the RG network layer^62,63^. For the RG model, two phase oscillators, whose phases are *ϕ*_r_ and *ϕ*_l_, were used, and each oscillator controls the corresponding hindlimb (*ϕ*_r_: right, *ϕ*_l_: left). Based on the network model^64^, where oscillators interact with each other, the oscillator phases are governed by the following dynamics

1 $$\begin{array}{c}{\dot{\varphi }}_{{\rm{r}}}=\omega -{K}_{\varphi }\,\sin \,({\varphi }_{{\rm{r}}}-{\varphi }_{{\rm{l}}}+\pi )\\ {\dot{\varphi }}_{{\rm{l}}}=\omega -{K}_{\varphi }\,\sin \,({\varphi }_{{\rm{l}}}-{\varphi }_{{\rm{r}}}-\pi )\end{array}$$

where *ω* is the basic oscillator frequency commonly used for the fast and slow side, and *K*_ϕ_ is the gain parameter to regulate anti-phase behavior between the oscillators; *ω* = 10.5 rad/s and *K*_ϕ_ = 7.5 were used. For the PF model, three activation pulses ${u}_{i}^{{\rm{CPG}}}$ (*i* = 1,2,3), represented by rectangular pulses (Fig. 2C), were used. The onset timing depends on the oscillator phase *ϕ* (subscript r or l is omitted), as given by

2 $${u}_{i}^{{\rm{C}}{\rm{P}}{\rm{G}}}(\varphi )=\{\begin{array}{c}1{\varphi }_{i}^{{\rm{S}}{\rm{t}}{\rm{a}}{\rm{r}}{\rm{t}}}\le \varphi \le {\varphi }_{i}^{{\rm{S}}{\rm{t}}{\rm{a}}{\rm{r}}{\rm{t}}}+{\rm{\Delta }}{\varphi }_{i}\\ 0\,{\rm{o}}{\rm{t}}{\rm{h}}{\rm{e}}{\rm{r}}{\rm{w}}{\rm{i}}{\rm{s}}{\rm{e}}\end{array}i=1,2,3$$

where ${\varphi }_{i}^{{\rm{Start}}}$ and Δ*ϕ*_*i*_ (*i* = 1, 2, 3) are the onset phase and duration of the activation, respectively. ${u}_{1}^{{\rm{CPG}}}$, ${u}_{2}^{{\rm{CPG}}}$, and ${u}_{3}^{{\rm{CPG}}}$ contribute to early extension, late extension, and flexion phases, respectively (Fig. 2D). The motoneuron command *u*_*m*_ of muscle *m* (*m* = IL, GM, VL, TA, SO, BF, and GC) is given by the linear combination of the activation pulses by

3 $${u}_{m}=\sum _{i=1}^{3}\,{w}_{m,i}\cdot {u}_{i}^{{\rm{CPG}}}(\varphi )\,$$

where *w*_*m*,*i*_ (*i* = 1, 2, 3) is the weighting coefficient. The control parameters for the pulses were determined based on the EMG data of rats^20,31^ and the comparison of the simulated locomotor behavior with the measured kinematic data from rats in the tied-belt condition. The muscle activation *a*_*m*_ is determined by

4 $${\tau }_{{\rm{act}}}\,{\dot{a}}_{m}+\{\frac{{\tau }_{{\rm{act}}}}{{\tau }_{{\rm{deact}}}}+(1-\frac{{\tau }_{{\rm{act}}}}{{\tau }_{{\rm{deact}}}}){u}_{m}\}{a}_{m}={u}_{m}$$

where *u*_*m*_ is the motor command determined by the nervous system model, and *τ*_act_ and *τ*_deact_ are the activation and deactivation time constants, respectively; *τ*_act_ = 44 ms and *τ*_deact_ = 70 ms were used.

Hodgkin-Huxley style neuron model showed that the phase of the neurons’ activity rapidly changed depending on external signals^59^, which suggests that the neural system has mechanism to quickly move the phase of neurons’ activity. Physiological studies have shown that stimulation of the peripheral nerve of the leg resets the rhythmic motor activities produced by the CPG during locomotion^65–68^. It has been reported that, when chronic spinal cats walking on a treadmill are intercepted so that their leg motions are forced to stop and then gradually move backward, the onset of the swing motion is observed when their hip angle exceeds a certain angle^37^. The trend is also observed in normal walking without the interception. Moreover, when the IL muscle, which is the flexor muscle of the hip, is stretched, and the triceps surae muscle, which is the extensor muscle of the ankle, is unloaded, ongoing extensor muscle activities stop, and flexor muscle activity is initiated^19^. These results suggest that the sensory information about the hip angle is used for the reset of the phase of muscle activities to the onset phase of the swing motion. The phase resetting by hip extension was used for the model of sensorimotor coordination. More specifically, when the hip joint angle exceeds a threshold angle, the afferent signal is sent to the RG model, and it resets the oscillator phase to the onset phase of the swing phase (${\varphi }_{3}^{{\rm{Start}}}$; see Fig. 2C,D). To incorporate this phase resetting mechanism based on our previous works^20,21^, the phase dynamics (1) were modified by

5 $$\begin{array}{ccc}{\dot{\varphi }}_{{\rm{r}}} & = & \omega -{K}_{\varphi }\,\sin ({\varphi }_{{\rm{r}}}-{\varphi }_{{\rm{l}}}+\pi )-({\varphi }_{{\rm{r}}}-{\varphi }^{{\rm{R}}{\rm{e}}{\rm{s}}{\rm{e}}{\rm{t}}})\delta (t-{t}_{{\rm{r}}}^{{\rm{H}}{\rm{i}}{\rm{p}}}-{\tau }^{{\rm{d}}{\rm{e}}{\rm{l}}{\rm{a}}{\rm{y}}})\\ {\dot{\varphi }}_{{\rm{l}}} & = & \omega -{K}_{\varphi }\,\sin \,({\varphi }_{{\rm{l}}}-{\varphi }_{{\rm{r}}}-\pi )-({\varphi }_{{\rm{l}}}-{\varphi }^{{\rm{R}}{\rm{e}}{\rm{s}}{\rm{e}}{\rm{t}}})\delta (t-{t}_{{\rm{l}}}^{{\rm{H}}{\rm{i}}{\rm{p}}}-{\tau }^{{\rm{d}}{\rm{e}}{\rm{l}}{\rm{a}}{\rm{y}}})\end{array}$$

where *δ*(·) is Dirac’s delta function, ${t}_{i}^{{\rm{Hip}}}$ (*i* = r, l) is the time when the hip angle exceeds the threshold angle, *ϕ*^Reset^(=${\varphi }_{3}^{{\rm{Start}}}$) is the reference phase, and *τ*^delay^ = 20 ms is the transfer delay of the afferent signal. A threshold angle of 136° was used so that the kinematic of the model with phase resetting was close to that without phase resetting in the split-belt configuration. The value of resetting is calculated by *ϕ*^Reset^−*ϕ*_*i*_ when $t={t}_{i}^{{\rm{Hip}}}+{\tau }^{{\rm{delay}}}(i={\rm{r}},{\rm{l}})$. Note that although phase resetting and interaction between the oscillators changed each oscillator frequency from the basic value *ω*, identical frequencies were achieved in steady locomotion even in the split-belt configuration.

## Electronic supplementary material

Supplementary information

Supplementary Movie S1

Supplementary Movie S2

Supplementary Movie S3

Supplementary Dataset S1

Supplementary Dataset S2
