# Supplementary material for: Adaptive hindlimb split-belt treadmill walking in rats by controlling basic muscle activation patterns via phase resetting
Source: Sci Rep. 2018 Nov 26;8:17341. doi: 10.1038/s41598-018-35714-8 (PMC6255885; doi:10.1038/s41598-018-35714-8)
Supplement: Supplementary file 1 — Supplementary information [file 41598_2018_35714_MOESM1_ESM.pdf]

Supplementary information

Adaptive hindlimb split-belt treadmill walking in rats by controlling basic muscle activation patterns via phase resetting

Soichiro Fujiki[1]*, Shinya Aoi[2], Tetsuro Funato[3], Yota Sato[3], Kazuo Tsuchiya[2], Dai Yanagihara[1]

[1] Department of Life Sciences, Graduate School of Arts and Sciences, The University of Tokyo, 3-8-1 Komaba, Meguro-ku, Tokyo 153-8902, Japan.

[2] Department of Aeronautics and Astronautics, Graduate School of Engineering, Kyoto University, Kyoto daigaku-Katsura, Nishikyo-ku, Kyoto 615-8540, Japan.

[3] Department of Mechanical Engineering and Intelligent Systems, Graduate School of Informatics and Engineering, The University of Electro-communications, 1-5-1 Chofugaoka, Chofu-shi, Tokyo 182-8585, Japan.

*** Corresponding author:**

Supplementary Method S1. Measurement of rats.

To measure the kinematic data of hindlimb motion, reflective markers were attached to the skin overlying the iliac crest, greater trochanter, knee joint, lateral malleolus, and fifth metatarsal head of both hindlimbs (Fig. 1B). The trajectories of markers were obtained using a 3D motion capture system (Qualisys, Gothenburg, Sweden) at a sampling rate of 500 Hz. The measured marker position data were low-pass filtered at 10 Hz with a second-order Butterworth filter, and the joint angles (hip, knee, and ankle) were calculated (Fig. 1B). All time series of the kinematic data were normalized by touchdown of the feet.

Supplementary Method S2. Musculoskeletal model and parameters.

Skeletal model. The skeletal model consists of seven rigid links representing the trunk and hindlimbs in two-dimensional space (sagittal plane) (Fig. 2A). The forelimbs are fixed on the trunk, and the tips are connected by a bar through viscoelastic elements. The hindlimbs consist of the thigh, shank, and foot, which are connected by rotational joints (hip, knee, and ankle). To simulate the split-belt treadmill environment, a belt model was used for each hindlimb. The foot contact with the belt was modeled using vertical viscoelastic elements and horizontal viscous elements. The equations of motion for the skeletal model were derived using Lagrangian equations.

Muscle model. The muscle model uses seven principal muscles for each hindlimb [iliopsoas (IL), gluteus maximus (GM), vastus lateralis (VL), tibialis anterior (TA), soleus (SO), biceps femoris (BF), and gastrocnemius (GC)] (Fig. 2B). The muscle tension F_m of muscle *m* (*m* = IL, GM, VL, TA, SO, BF, and GC) is given by

$$\boldsymbol{F_m} = \boldsymbol{F_m^{\mathrm{max}}}\left(\boldsymbol{a_m} \cdot \boldsymbol{F_m^{\mathrm{l}}} \cdot \boldsymbol{F_m^{\mathrm{v}}} + \boldsymbol{F_m^{\mathrm{p}}}\right) \quad \textbf{(S1)}$$

where F_m^{max} is the maximum muscle tension, a_m is the muscle activation, F_m^{l} and F_m^{v} are the force-length and force-velocity relationships, and F_m^{p} is the passive component. The moment arms of the muscles around the joints are constant, regardless of the joint angles.

Physical parameter. The physical parameters of the musculoskeletal model (Figs. 2A and B) were as follows. The masses of the trunk, thigh, shank, and foot were 153, 7.42, 3.99, and 2.15 g, respectively. The total mass was 180.1 g. The lengths of the trunk along the cranial-caudal direction, the trunk along the anterior-posterior direction, and the thigh, shank, and foot were 72.1, 18.4, 24.6, 36.2, and 19.6 mm, respectively. The moments of inertia around the center of mass of the trunk, thigh, shank, and foot were 4.52×10^5, 1.46×10^3, 665, and 191 g mm^2, respectively. The height of the bar was set at 100 mm. F_m^{max} of IL, GM, VL, TA, SO, BF, and GC were 22.6, 40.5, 27.5, 5.88, 5.00, 4.40, and 6.41 N,

respectively. The moment arms of IL, GM, VL, TA, SO, BF at the hip side, BF at the knee side, GC at the knee side, and GC at the ankle side were 5.94, 2.53, 5.33, 6.77, 7.97, 3.33, 16.6, 5.64, and 7.77 mm, respectively.

Control parameter. In the simulation study, the treadmill configuration was suddenly changed from slow-tied to split-belt configurations to investigate the adaptive locomotor behavior of the rat model in the split-belt configuration. The control parameters of the pulses were determined based on the EMG data of rats [20,31] so that the model walked stably irrespective of the use of phase resetting in the ST: $\phi_1^{\mathrm{Start}} = 0.50$ rad, $\Delta\phi_1 = 0.56$ rad, $\phi_2^{\mathrm{Start}} = 1.85$ rad, $\Delta\phi_2 = 2.00$ rad, $\phi_3^{\mathrm{Start}} = 3.85$ rad, $\Delta\phi_3 = 0.87$ rad, $w_{\mathrm{IL3}} = 0.23$, $w_{\mathrm{GM1}} = 0.33$, $w_{\mathrm{GM2}} = 0.10$, $w_{\mathrm{VL1}} = 0.30$, $w_{\mathrm{VL2}} = 0.17$, $w_{\mathrm{TA3}} = 0.17$, $w_{\mathrm{SO1}} = 0.55$, $w_{\mathrm{SO2}} = 0.18$, $w_{\mathrm{BF1}} = 0.35$, $w_{\mathrm{BF2}} = 0.23$, $w_{\mathrm{BF3}} = 0.22$, $w_{\mathrm{GC1}} = 0.35$, and $w_{\mathrm{GC2}} = 0.08$. The parameters were the same for all belt speed conditions.

Supplementary Table S1. Results of ANOVA for four locomotion parameters.

Parameters	Mean ± SE in period				F value	p value
	ST1	FT	ST2	SB		
Relative phase						
1.5x	3.26±0.175	3.14±0.105	3.36±0.199	2.53±0.129	F(3,56)=5.75	p=0.0017*
1.7x	3.14±0.228	3.21±0.205	3.08±0.219	2.12±0.132	F(3,44)=6.53	p=0.0009*
2.0x	3.12±0.159	2.96±0.154	3.06±0.215	2.30±0.134	F(3,40)=5.07	p=0.0045*
Duty factor						
1.5x	1.03±0.056	1.00±0.036	1.05±0.060	0.77±0.046	F(3,56)=6.92	p=0.0005*
1.7x	0.99±0.035	0.99±0.059	0.97±0.029	0.63±0.033	F(3,44)=19.5	p<<0.01*
2.0x	1.03±0.038	0.96±0.041	0.99±0.022	0.65±0.045	F(3,40)=21.7	p<<0.01*
Stride length						
1.5x	1.01±0.047	0.95±0.030	1.00±0.036	0.93±0.052	F(3,56)=0.80	p=0.50
1.7x	1.02±0.044	1.00±0.039	0.96±0.035	0.93±0.055	F(3,44)=0.78	p=0.51
2.0x	1.04±0.040	1.02±0.032	0.99±0.018	1.01±0.057	F(3,40)=0.35	p=0.79
Step length						
1.5x	0.98±0.077	0.97±0.048	1.05±0.079	0.73±0.044	F(3,56)=4.70	p=0.0054*
1.7x	1.07±0.110	1.09±0.040	1.05±0.078	0.70±0.054	F(3,44)=6.17	p=0.0014*

2.0x	0.97±0.052	0.98±0.053	0.98±0.052	0.72±0.052	F(3,40)=6.33	p=0.0013*

Numerical and statistical parameters illustrated in Fig. 4 are shown. ST: slow-tied configuration. FT: fast-tied configuration. SB: split-belt configuration. * p<0.05.

Supplementary Table S2. Results of ANOVA for liftoff and touchdown timings.

Parameters	Mean ± SE in period				F value	p value
	ST1	FT	ST2	SB		
Liftoff phase						
1.5x	3.64±0.088	3.14±0.113	3.77±0.145	2.95±0.157	F(3,56)=9.77	p<<0.01*
1.7x	3.71±0.126	3.08±0.170	3.77±0.113	2.89±0.201	F(3,44)=7.93	p=0.0002*
2.0x	3.77±0.082	2.95±0.132	3.83±0.082	2.70±0.138	F(3,40)=27.1	p<<0.01*
Touchdown phase						
1.5x	3.02±0.119	3.14±0.069	2.95±0.138	3.08±0.132	F(3,56)=0.38	p=0.77
1.7x	3.14±0.132	3.02±0.101	3.14±0.163	3.14±0.151	F(3,44)=0.17	p=0.92
2.0x	3.27±0.145	3.27±0.101	3.14±0.195	3.08±0.107	F(3,40)=0.21	p=0.89

Numerical and statistical parameters illustrated in Fig. 5 are shown. ST: slow-tied configuration. FT: fast-tied configuration. SB: split-belt configuration. * p<0.05.

Supplementary Table S3. Results of ANOVA for hip joint angles at liftoff.

Leg	Mean ± SE in period				F value	p value
	ST1	FT	ST2	SB		
Fast leg						
1.5x	109±3.68	108±3.47	108±3.70	107±3.62	F(3,48)=0.029	p=0.99
1.7x	114±3.10	113±3.73	113±3.35	113±3.68	F(3,52)<<0.01	p=1.00
2.0x	112±5.49	113±6.02	113±5.77	115±5.61	F(3,40)=0.035	p=0.99
Slow leg						
1.5x	115±4.43	116±4.04	116±3.93	113±406	F(3,48)=0.141	p=0.93
1.7x	115±3.37	113±3.47	114±3.38	114±3.72	F(3,52)=0.060	p=0.98
2.0x	115±6.06	113±6.09	116±6.39	113±6.12	F(3,40)=0.051	p=0.98

Numerical and statistical parameters illustrated in Fig. 6 are shown. ST: slow-tied configuration. FT: fast-tied configuration. SB: split-belt configuration. * p<0.05.

Supplementary Table S4. Experimental conditions for each session.

Session No.	Rat ID	Speed ratio	Fast leg	Analysis
1	R1406	1.5x	Right	Both
2	R1406	1.5x	Left	Both
3	R1406	1.5x	Right	1
4	R1407	1.5x	Left	Both
5	R1407	1.5x	Right	Both
6	R1409	1.5x	Left	Both
7	R1409	1.5x	Right	Both
8	R1410	1.5x	Left	Both
9	R1412	1.5x	Right	Both
10	R1412	1.5x	Left	Both
11	R1412	1.5x	Right	Both
12	R1501	1.5x	Right	1
13	R1502	1.5x	Right	Both
14	R1505	1.5x	Right	Both
15	R1506	1.5x	Right	Both
16	R1407	1.7x	Right	Both
17	R1409	1.7x	Right	Both
18	R1411	1.7x	Right	2
19	R1413	1.7x	Right	2
20	R1501	1.7x	Left	Both
21	R1501	1.7x	Right	Both
22	R1502	1.7x	Left	Both
23	R1502	1.7x	Left	Both
24	R1502	1.7x	Left	Both
25	R1502	1.7x	Right	Both
26	R1503	1.7x	Left	Both
27	R1504	1.7x	Left	Both
28	R1505	1.7x	Left	Both
29	R1506	1.7x	Right	Both
30	R1406	2.0x	Left	Both
31	R1407	2.0x	Left	Both
32	R1409	2.0x	Left	Both
33	R1410	2.0x	Left	Both
34	R1411	2.0x	Left	Both

35	R1412	2.0x	Left	Both
36	R1413	2.0x	Left	Both
37	R1503	2.0x	Right	Both
38	R1504	2.0x	Left	Both
39	R1504	2.0x	Right	Both
40	R1505	2.0x	Left	Both

Speed ratio shows the ratio between the belt speeds (fast/slow) used in the split-belt configuration. Fast leg represents the fast side during the split-belt configuration. Analysis represents the measured data used for the statistical analysis shown in Table 1 (1; analysis of locomotion parameters and transition timings, 2; analysis of hip angle at liftoff, or both).

Supplementary Figure S1. Oscillator phases of two legs.

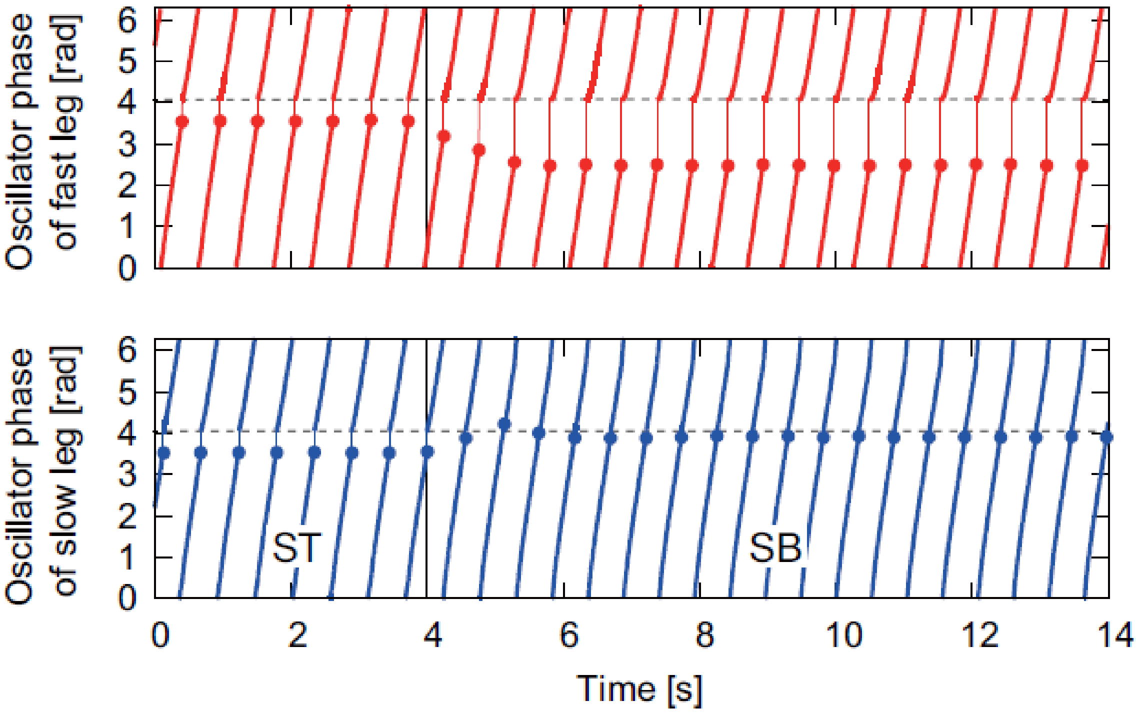

Time profiles of the oscillator phases (ϕ_l, ϕ_r) in the 2.0x condition are shown. The circle mark represents the phase value when the phase resetting occurred. The dashed line represents the phase value just after phase resetting. The vertical lines show the transition time from the slow-tied to split-belt configuration. Because phase resetting deviated the relative phase between the oscillators from antiphase, the slopes changed just after the phase resetting of the ipsilateral and contralateral sides due to the interaction between the oscillators. More specifically, the fast leg reached sooner the critical hip angle in the split-belt configuration than in the slow-tied configuration, which explains the larger phase resetting in the split-belt configuration. Just after the phase resetting in the fast leg in the

split-belt configuration, because the interaction term of the fast leg had a negative value and that of slow leg had a positive value from equation (1), the oscillator of the fast leg slowed down and that of the slow leg speeded up. As a result, the two oscillators had the same gait cycle duration. Slow-tied configuration: ST. Split-belt configuration: SB.

Supplementary Movie S1. Simulated locomotor behavior of the rat model with phase resetting.
This file shows the simulated locomotor behavior of our rat model during the slow-tied and split-belt configurations (2.0x condition) with phase resetting.

Supplementary Movie S2. Simulated locomotor behavior of the rat model without phase resetting (2.0x condition).
This file shows the simulated locomotor behavior of our rat model during the slow-tied and split-belt configurations (2.0x condition) without phase resetting.

Supplementary Movie S3. Simulated locomotor behavior of the rat model without phase resetting (1.7x condition).
This file shows the simulated locomotor behavior of our rat model during the slow-tied and split-belt configurations (1.7x condition) without phase resetting.

Supplementary Dataset S1. Measured data.
A compressed archive file (zip) contains seven folders, and each folder contains data files in comma-separated values format (csv) and descriptive text about the data (txt). Files in the 'RelativePhase', 'DutyFactor', 'StrideLength', and 'StepLength' folders are the data of relative phase, duty factor, stride length, and step length, respectively, in each session. Files in the 'TouchdownLiftoff' folder are the data for the touchdown phase of the slow leg and the liftoff phase of the fast leg in each session. Files in the 'HipAngle' folder are the data for the hip angle at liftoff in each session. Files in the 'RepresentativeData' folder are the data used for the representative results of the measurements (Figs. 3 and 4).

Supplementary Dataset S2. Simulated Data.
A compressed archive file (zip) contains six folders [three belt speed conditions (1.5x, 1.7x, and 2.0x) with and without phase resetting], and each folder contains data files in comma-separated values format (csv) and a descriptive text about the data (txt).
